# Proteoglycan-4 is an essential regulator of synovial macrophage polarization and inflammatory macrophage joint infiltration

**DOI:** 10.1186/s13075-021-02621-9

**Published:** 2021-09-14

**Authors:** Marwa Qadri, Gregory D. Jay, Ling X. Zhang, Tannin A. Schmidt, Jennifer Totonchy, Khaled A. Elsaid

**Affiliations:** 1 Department of Pharmacology, College of Pharmacy, Jazan University, Jazan, 82826 Kingdom of Saudi Arabia; 2 Department of Emergency Medicine, Rhode Island Hospital, Providence, RI USA; 3 Biomedical Engineering Department, School of Dental Medicine, University of Connecticut, Farmington, CT USA; 4 Department of Biomedical and Pharmaceutical Sciences, Chapman University School of Pharmacy, Rinker Health Sciences Campus, 9401 Jeronimo Road, Irvine, CA 92618 USA

**Keywords:** PRG4, Synovial macrophages, Bone-marrow-derived macrophages, Synovial hyperplasia

## Abstract

**Abstract:**

**Background:**

Synovial macrophages perform a multitude of functions that include clearance of cell debris and foreign bodies, tissue immune surveillance, and resolution of inflammation. The functional diversity of macrophages is enabled by distinct subpopulations that express unique surface markers. Proteoglycan-4 (PRG4) is an important regulator of synovial hyperplasia and fibrotic remodeling, and the involvement of macrophages in PRG4’s synovial role is yet to be defined. Our objectives were to study the PRG4’s importance to macrophage homeostatic regulation in the synovium and infiltration of pro-inflammatory macrophages in acute synovitis and investigate whether macrophages mediated synovial fibrosis in *Prg4* gene-trap (*Prg4*^*GT/GT*^) murine knee joints.

**Methods:**

Macrophage phenotyping in *Prg4*^*GT/GT*^ and *Prg4*^*+/+*^ joints was performed by flow cytometry using pan-macrophage markers, e.g., CD11b, F4/80, and surface markers of M1 macrophages (CD86) and M2 macrophages (CD206). Characterizations of the various macrophage subpopulations were performed in 2- and 6-month-old animals. The expression of inflammatory markers, IL-6, and iNOS in macrophages that are CD86+ and/or CD206+ was studied. The impact of *Prg4* recombination on synovial macrophage populations of 2- and 6-month-old animals and infiltration of pro-inflammatory macrophages in response to a TLR2 agonist challenge was determined. Macrophages were depleted using liposomal clodronate and synovial membrane thickness, and the expression of fibrotic markers α-SMA, PLOD2, and collagen type I (COL-I) was assessed using immunohistochemistry.

**Results:**

Total macrophages in *Prg4*^*GT/GT*^ joints were higher than *Prg4*^*+/+*^ joints (*p<0.0001*) at 2 and 6 months, and the percentages of CD86+/CD206− and CD86+/CD206+ macrophages increased in *Prg4*^*GT*/GT^ joints at 6 months (*p<0.0001*), whereas the percentage of CD86−/CD206+ macrophages decreased (*p<0.001*). CD86+/CD206− and CD86+/CD206+ macrophages expressed iNOS and IL-6 compared to CD86−/CD206+ macrophages (*p<0.0001*). *Prg4* re-expression limited the accumulation of CD86+ macrophages (*p<0.05*) and increased CD86−/CD206+ macrophages (*p<0.001*) at 6 months. *Prg4* recombination attenuated synovial recruitment of pro-inflammatory macrophages in 2-month-old animals (*p<0.001*). Clodronate-mediated macrophage depletion reduced synovial hyperplasia, α-SMA, PLOD2, and COL-I expressions in the synovium (*p<0.0001*).

**Conclusions:**

PRG4 regulates the accumulation and homeostatic balance of macrophages in the synovium. In its absence, the synovium becomes populated with M1 macrophages. Furthermore, macrophages exert an effector role in synovial fibrosis in *Prg4*^*GT/GT*^ animals*.*

**Supplementary Information:**

The online version contains supplementary material available at 10.1186/s13075-021-02621-9.

## Background

The synovium is a soft tissue comprised of a surface layer, the intima, and an underlying subintima [1, 2]. The synovial intima is 1–3-cell-layer thick, with two cell types: fibroblast-like synoviocytes and macrophages [1, 2]. The subintima is less cellular with significant collagen type I content, microvascular blood, and lymph vessels [2]. Synovial macrophages comprise distinct subsets of heterogenous cell populations that perform various functions including clearance of cell debris and foreign bodies, tissue immune surveillance, and resolution of inflammation [3–5]. The synovial microenvironment regulates macrophage heterogeneity as it controls the polarization of macrophages on a spectrum of pro-inflammatory and anti-inflammatory functions [6–9]. At one end of the spectrum is the M1 macrophage phenotype, with a pro-inflammatory role mediated by the production of high levels of interleukin-1 beta (IL-1β), tumor necrosis factor alpha (TNF-α), IL-6, inducible nitric oxide synthase (iNOS) expression specifically in murine macrophages, and generation of reactive oxygen species [10–12]. At the opposite end is the M2 macrophage phenotype, which is characterized by the production of anti-inflammatory cytokines; IL-10, interleukin-1 receptor antagonist (IL-1Ra), and transforming growth factor beta (TGF-β) [9, 10, 13]. M1 macrophages have been associated with the exacerbation of synovial inflammation, and related joint destruction in OA and RA [12, 14], while the population of the synovium by M2 macrophages was associated with resolution of joint inflammation [15].

Proteoglycan-4 (PRG4) is a mucinous glycoprotein secreted by fibroblast-like synoviocytes and superficial zone chondrocytes [16, 17]. PRG4 functions as a boundary lubricant between apposed cartilage surfaces, which prevents mitochondrial dysregulation and superficial zone chondrocyte apoptosis [18]. Biologically, PRG4 engages a number of receptors including CD44 and the Toll-like receptors 2 and 4 (TLR2 and TLR4) [19–21]. PRG4-CD44 interaction inhibits nuclear factor kappa B (NF-κB) nuclear translocation in human synovial fibroblasts and attenuates IL-1β-induced synoviocyte proliferation and expression of matrix-degrading enzymes [22]. PRG4 regulates synovial fibroblast to myofibroblast transition and reduces the expression of fibrotic markers*:* alpha smooth muscle actin (α-SMA) and collagen type I [23, 24]. PRG4 also suppresses the activation of TLR2 and TLR4 receptors by DAMPs in OA synovial fluid aspirates [21]. Synovial hyperplasia and fibrosis, that progressed with age, are evident in murine knee joints lacking *Prg4* expression [23, 25, 26]. The biological role of PRG4 helps explain its disease-modifying activity in rodent and pig models of posttraumatic osteoarthritis (PTOA), where PRG4 reduced cartilage degeneration and enhanced its repair [27, 28]. While PRG4’s effects on synovial fibroblasts have been characterized, its contribution to synovial macrophage polarization on the M1-M2 spectrum remains largely unknown. Preliminary evidence suggests that PRG4 may influence macrophage responsiveness as peritoneal macrophages from *Prg4* knockout mice secreted higher levels of IL-1β following TLR stimulation compared to peritoneal macrophages from *Prg4* expressing mice [29].

Synovitis is a significant and common finding in patients with knee OA and is an independent predictor of radiographic OA progression [30]. Synovitis is characterized by synovial hyperplasia, subintimal fibrosis, and neovascularization [31]. Macrophages and T cells are the predominant immune cells encountered in the OA synovium, and most inflammatory cytokines produced in synovitis are attributed to activated synovial macrophages [31]. Synovial fibrosis is a maladaptive resolving response to chronic synovitis and is characterized by increased cross-linked collagen I deposition, due to a combination of enhanced collagen production and PLOD2-mediated crosslinking, and transition of synovial fibroblasts to activated myofibroblasts, characterized by de novo α-SMA expression [23, 24]. Synovial fibrosis has an immune-mediated etiology, and it is hypothesized that DAMPs trigger TLR-mediated activation of synovial macrophages and subsequent release of TGF-β which in turn drives the fibrotic remodeling of the synovium [32].

In this study, we aimed to investigate the role of PRG4 in regulating macrophage phenotype polarization and accumulation in synovial tissues and the recruitment of pro-inflammatory macrophages in response to acute synovitis, and to evaluate whether chronic synovitis and synovial fibrosis in *Prg4* null joints are mediated by an enhanced polarization towards a pro-inflammatory macrophage phenotype. In the course of our investigation, we studied synovium-resident macrophages (SRMs) and total macrophages in isolated synovial tissues using differential and overlapping markers. We also investigated the recruitment of pro-inflammatory macrophages that have originated from circulating monocytes in response to a TLR2 agonist intra-articular challenge. We hypothesized that PRG4 maintains synovial macrophage homeostasis, and in its absence, macrophages shift towards an M1 pro-inflammatory phenotype resulting in chronic synovitis and fibrotic remodeling.

## Methods

### Animals and study overview

The *Prg4* gene-trap (*Prg4*^*GT*^) mouse (stock no. 025740, JAX, USA) is born lacking *Prg4* expression which can be restored via CRE-mediated recombination [26]. The *Prg*^*GT/GT*^ animal displays significant synovial hyperplasia and subintimal fibrosis and recombination appeared to attenuate these changes [23]. In this study, *Prg4* recombination (*Prg4*^*GTR/GTR*^) occurred in 3-week-old animals via intraperitoneal injection of tamoxifen (0.1mg/g in 100μL corn oil vehicle) daily for 10 days, using vehicle-administered gene-trap animals as controls. We have also used *Prg4*^*+/+*^ animals (stock # 101045, JAX; B6/129S background). Animals were studied at 2 or 6 months of age and depletion of macrophages was performed when animals were 4-month-old, and we included randomly assigned litter and age-matched males and females in our experimental groups. The number of animals per group was based on our previous analysis of the effect of *Prg4* recombination on synovial fibrosis using alpha-smooth muscle actin (α-SMA) staining as our primary endpoint [23]. Our studies were approved by the IACUC committee at Chapman University. All experiments were performed according to established guidelines and regulations.

We studied SRMs in 2- and 6-month-old *Prg4*^*GT/GT*^ and *Prg4*^*+/+*^ knee joints. We identified SRMs using CD68—a tissue-resident macrophage marker—CD11b, and F4/80 [5, 14]. SRMs were identified as CD68+ CD11b+ F4/80+ MHC class II−. We also studied the expression of CX3CR1 receptor in SRMs of *Prg4*^*GT/GT*^ and *Prg4*^*+/+*^ joints as CX3CR1+ SRMs were recently shown to play an anti-inflammatory homeostatic role [5]. Total macrophages in the synovium were identified as CD11b+ F4/80+ MHC class II− and a combination of either CD86+/CD206−, CD86+/CD206+ or CD86−/CD206+. In characterizing these macrophages, we utilized CD86 (an M1 marker) [33] and CD206 (an M2 marker) [34] to provide us with an understanding of the balance between the M1 and M2 phenotypes under different experimental conditions. We confirmed the pro-inflammatory phenotype of CD86+ macrophages by iNOS and IL-6 staining, and the anti-inflammatory phenotype of M2 macrophages by arginase-1 (Arg-1) staining [35]. We also studied the impact of *Prg4* recombination on total macrophage numbers and the balance between CD86+ and CD206+ macrophages. To further characterize the role of PRG4 in regulating the recruitment of pro-inflammatory macrophages, we induced synovitis in *Prg4*^*GT/GT*^ and *Prg4*^*GTR/GTR*^ knee joints using Pam3CSK4 (Invivogen, USA), a TLR2 agonist [36]. We identified newly infiltrated pro-inflammatory macrophages, based on MHC class II and Ly-6C positivity as CD11b+ F4/80+ MHC class II+ Ly-6C+ CD86+ [14, 37]. We also depleted macrophages in *Prg4*^*GT/GT*^ joints using intra-articular (IA) clodronate liposomes and confirmed macrophage depletion by CD11b immunoprobing in the synovial tissues of these animals. We selected an intermediate endpoint, day 15 following clodronate treatment initiation, to evaluate changes to CD86+ and/or CD206+ macrophages in synovial tissues. Using immunohistochemistry, we also studied the impact of macrophage depletion at day 30 on synovial hyperplasia and fibrosis in *Prg4*^*GT/GT*^ joints. Finally, we supplemented our in vivo studies with in vitro studies using murine M2a BMDMs. Our rationale to study M2a macrophages was based on the current understanding that M2 macrophages are the predominant population in the synovium in the absence of an inflammatory stimulus. We used an established differentiation protocol to generate M2a BMDMs and tested whether rhPRG4 treatment modulated the secretion of cytokines and chemokines in TLR2 agonist-stimulated M2a BMDMs. We focused our efforts on the M2a subtype given its role in resolving inflammation and promoting wound healing [9, 10]. An overview of the experimental approach for our in vivo studies is shown in Supplementary Figure 1.

### Immunophenotyping of various macrophage populations

Joint capsular tissues were isolated, pooled from two knee joints to generate an independent sample, and digested using collagenase-D (2mg/mL) (Sigma Aldrich, USA) + 1μL RNase-free DNase I (1U/μL) (ThermoFisher Scientific, USA) in Hanks’ balanced salt solution (1mL) for 1h at 37^o^C. Cell suspensions were then filtered through a 40-μm nylon mesh (Sigma Aldrich), and cells were pelleted at 3000 rpm for 5 min. Cells were stained with a viability dye (Zombie Violet; BioLegend, USA) and fluorochrome-conjugated antibodies as described below. The numbers of macrophages of interest were estimated using Precision Counting beads (BioLegend). Our panel was designed using the BD Horizon Guided Panel Solution tool, and the positivity threshold for marker staining was determined using fluorescence minus one (FMO) panel controls. Fluorochrome-conjugated antibodies included Alexa Fluor 488-anti-iNOS and PE-anti-Arg-1 (ThermoFisher Scientific), APC-Cy7-anti-CD11b, FITC-anti-Ly-6C, PE-Cy7 anti-CD86 and PerCP-Cy5.5 anti-I-A/I-E (MHC class II) (BD Biosciences, USA), PE-anti-CD68, Brilliant Violet 510-anti-F4/80, Alexa fluor 488-anti-CX3CR1, PE-anti-IL-6, and APC-anti-CD206 (BioLegend) and were used at recommended dilutions. Cells were suspended in FACS blocking buffer (0.5% BSA + 2% FBS in PBS) for 10 min on ice and then incubated with the antibody mix in FACS staining buffer (0.5% BSA + 0.05% sodium azide in PBS) for 20 min on ice. Intracellular staining of Arg-1, CD68, IL-6, and iNOS proteins was performed following surface marker staining. Cells were suspended in BD Cytofix/Cytoperm buffer containing 4.2% formaldehyde with fluorochrome-conjugated antibodies diluted according to manufacturer’s recommendations and incubated for 20 min on ice. Subsequently, cells were washed twice with FACS washing buffer followed by flow cytometric analysis (BD FACSAria). Flow cytometry plots and analyses were generated using Flow Jo® software (BD Biosciences). We performed two technical replicates for each independent biological sample, and the analysis output was the average of the two technical replicates.

### Generation of M2a BMDMs

The bone marrows of the femurs and tibias of 2-month-old *Prg4*^*GT/GT*^ and *Prg4*^*+/+*^ animals were isolated, and BMDMs were generated as described [36, 38]. On day 7, M2a BMDMs were induced by IL-4 + IL-13 (20 ng/mL for both cytokines; R&D Systems) for 24h [38].

### Target gene expression and analysis of secreted cytokines and chemokines by Prg4^GT/GT^ BMDMs following TLR2 stimulation ± rhPRG4

M2a BMDMs from 2-month-old *Prg4*^*GT/GT*^ and *Prg4*^*+/+*^ joints (500,000 cells per well) were treated with Pam3CSK4 (300ng/mL) or cultured as untreated control for 24h, in serum-free DMEM/F12 media, followed by RNA isolation, cDNA synthesis, and quantitative PCR as described [36]. The cycle threshold (Ct) values of genes of interest (*Nos2*; *gene symbol for iNOS*, *IL-1β*, *IL-6*, *IL-10*, and *TGF-β*) were normalized to the Ct value of *GAPDH* in the same sample, and relative expression was calculated using the 2^-ΔΔCt^ method [39]. The following primers and probes were used: *Nos2* (Mm00440502_m1), *IL-1β* (Mm00434228_m1), *IL-6* (Mm00446190_m1), *IL-10* (Mm01288386_m1), *TGF-β* (Mm01178820_m1), and *GAPDH* (Mm99999915_g1) (all available from ThermoFisher Scientific). M2a BMDMs from *Prg4*^*GT/GT*^ animals were treated with Pam3CSK4 ± rhPRG4 [40] (200μg/mL) for 24h followed by gene expression studies and profiling of secreted cytokines and chemokines using Proteome Profiler Mouse Cytokine Array Kit, Panel A (R&D Systems). Signal intensities of protein spots were quantified using Image J® and normalized to signal intensities of reference spots. Data are presented as mean ± S.D. of integrated pixel densities from three independent experiments.

### IA administrations of Pam3CSK4, vehicle, rhPRG4, or PBS

IA injections were performed in the knee joints of 2-month-old mice under inhaled isoflurane gas anesthesia (5% induction and 2–3% maintenance). The mouse skin was shaved and cleansed with a topical antiseptic. IA injections were performed under a magnifying lens, and a total of 10μL was delivered through the patellar tendon. The effects of IA Pam3CSK4 (3μg in 10 μL) were compared to vehicle (sterile water; 10 μL) in *Prg4*^*GT/GT*^ and *Prg4*^*GTR/GTR*^ animals. IA rhPRG4 (1mg/mL; 10μL) or phosphate-buffered saline (PBS) (10μL) injections were performed in *Prg4*^*GT/GT*^ animals, and analysis of CD11b+ F4/80+ MHC class II− CD86+ iNOS+ and CD11b+ F4/80+ MHC class II− CD86+ IL-6+ macrophages was conducted as described above at 24h.

### Macrophage depletion using clodronate liposomes and its impact on synovial fibrosis in Prg4^GT/GT^ knee joints

*Prg4*^*GT/GT*^ joints were injected with clodronate liposomes (10μL; 18.4 mM) or PBS liposomes (10μL) (Encapsula Nanosciences, USA) twice weekly for 2 weeks, and on day 15 following treatment initiation, we quantified the percentages of CD11b+ F4/80+ MHC class II− CD86+ or CD86−/CD206+ macrophages and the number of CD86+ macrophages as described above. IA clodronate or PBS liposomes injections were conducted as described above. In a parallel study, *Prg4*^*GT/GT*^ joints were treated with clodronate, or PBS liposomes as described above and the joints were harvested on day 30 following treatment initiation, and age-matched *Prg4*^*+/+*^ joints (4 months) were used as controls. The joints were decalcified, paraffin-embedded, and sectioned. Sections were stained with hematoxylin & eosin (H&E), and synovial membrane thickness was determined using the mean of five thickness measurements per specimen. Alternatively, sections were permeabilized with 0.1% triton x100 for 10 min followed by blocking with staining buffer for 20 min. Sections were incubated with rabbit polyclonal antibodies against α-SMA (Abcam, USA), procollagen-lysine-2-oxygultarate-5-dioxygenase 2 (PLOD2) (Abcam), collagen I (COL-I) (Abcam), and CD11b (Novus, USA) (1:100 dilutions for all antibodies) overnight at 4^o^C. Following washing with PBS, sections were incubated with Cy3-goat anti-rabbit IgG at 1:200 dilution for 1h at room temperature and imaged using a confocal microscope (Olympus BX 51). Regions of interest in synovia of different animals were defined, and integrated OD (Lum*μm^2^) values were computed and compared across groups.

### Statistical analyses

Statistical analyses were conducted as we previously described [23]. We used unpaired, paired Student’s *t* test, and one-way or two-way ANOVAs followed by Tukey’s post hoc tests, with statistical significance set at *p<0.05*. The specific test type is stated in figure legends.

## Results

*The tissue-resident fraction of macrophages was reduced in Prg4* ^*GT/GT*^ *synovia, and total macrophages liberated from synovial tissue digestion exhibited an age-dependent shift to a predominantly CD86+ pro-inflammatory phenotype and in vivo rhPRG4 treatment reduced inflammatory markers’ expression in this specific macrophage population.*

**Fig. 1:**
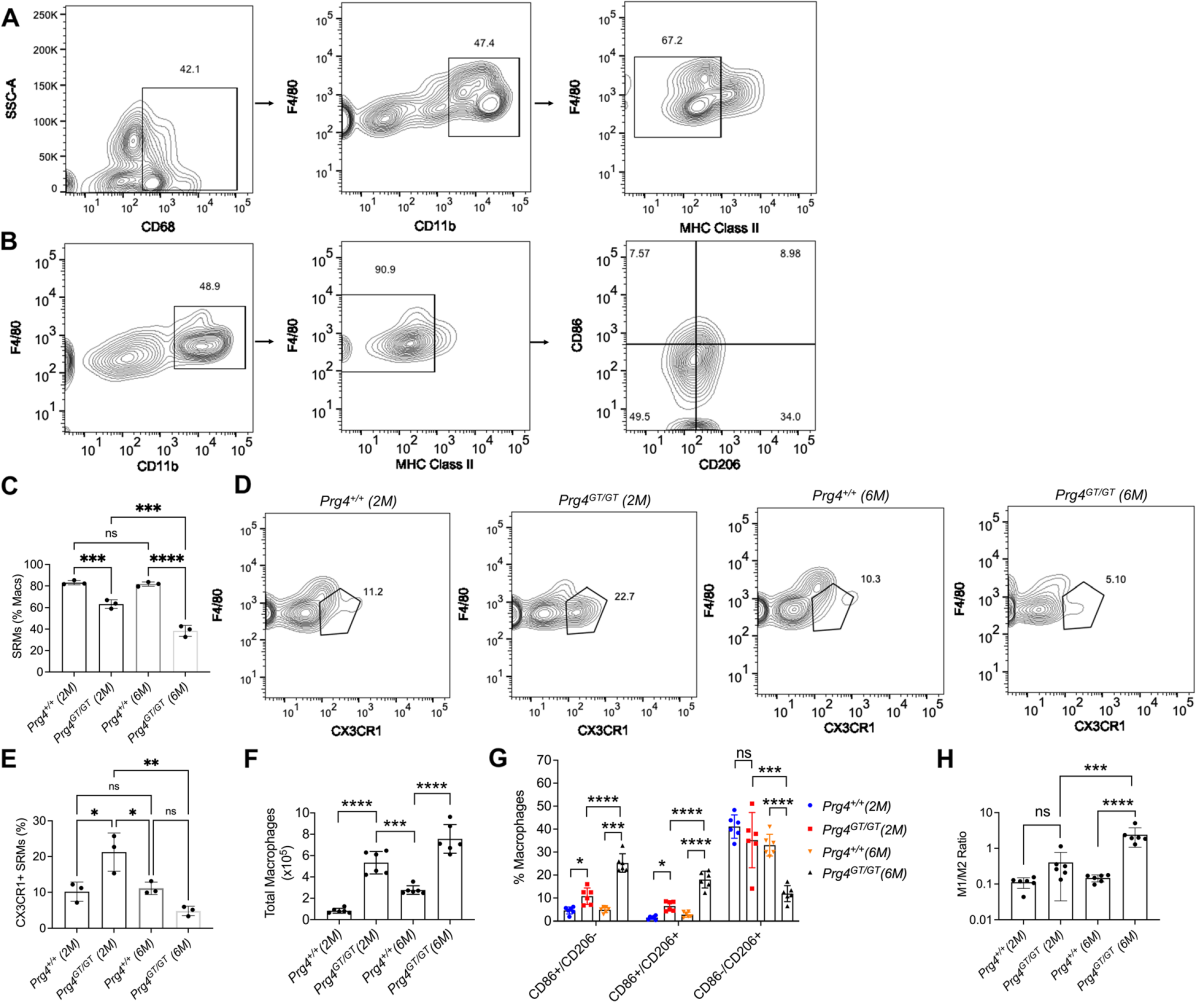
Analysis of synovium-resident macrophages (SRMs) in the knee joints of 2- and 6-month-old *Prg4* gene trap (*Prg4*^*GT/GT*^) and age-matched wildtype (*Prg4*^*+/+*^) mice and comparisons of total macrophages isolated from synovial tissues from both genotypes. SRMs were identified as CD68+ CD11b+ F4/80+ MHC class II− and expressed as % of total macrophages. The percentages of CX3CR1+ SRM subpopulation were analyzed as a function of age and genotype. Total macrophages were determined in cell suspensions of digested joint capsule tissues as described in the “Methods” section (CD11b+ F4/80+ MHC class II− CD86+ and/or CD206+). CD86 is a marker of inflammatory M1 macrophages while CD206 is a marker of anti-inflammatory M2 macrophages. In our flow cytometry analyses, singlets were initially gated using a combination of forward scatter height (FSC-H) and forward scatter area (FSC-A) and viable cells were subsequently identified using a viability dye (Zombie Violet) as shown in Supplementary Figure 2. Three animals per group were used to study SRMs (two males and one female), and six animals per group were used to study total synovial macrophages (balanced between males and females). In all experiments, independent biological samples were analyzed in duplicates, and the averages of both technical replicates were included in the analysis. We compared the ratio of M1 (CD11b+ F4/80+ MHC class II− CD86+/CD206−) and M2 (CD11b+ F4/80+ MHC class II− CD86−/CD206+) macrophages in synovial tissues across all experimental groups. Statistical analyses of SRMs, total macrophages, and percentages of CD86+ and/or CD206+ macrophages were performed using Student’s *t* test and one-way and two-way ANOVA, respectively. Tukey’s post hoc test was used in one- and two-way ANOVAs. ns non-significant; *p<0.05; **p<0.01; ***p<0.001; and ****p<0.0001. **A G**ating strategy was utilized to identify SRMs. **B** Gating strategy utilized to identify total macrophages in digested synovial tissues. **C** 2- and 6-month-old *Prg4*^*GT/GT*^ joints contained lower percentages of SRMs compared to age-matched *Prg4*^*+/+*^ joints. **D** Representative flow cytometry contour plots showing CX3CR1+ SRMs from 2- and 6-month-old *Prg4*^*GT/GT*^ and *Prg4*^*+/+*^ animals. **E** Percentage of CX3CR1+ SRMs in *Prg4*^*GT/GT*^ joints declined as animals aged. **F** 2- and 6-month-old *Prg4*^*GT/GT*^ synovial tissues contained higher numbers of total macrophages than age-matched *Prg4*^*+/+*^ synovia. **G** *Prg4*^*GT/GT*^ joints had higher percentages of CD86+/CD206− and CD86+/CD206+ macrophages (2 and 6 months) and a lower percentage of CD86−/CD206+ macrophages (6 months) compared to age-matched *Prg4*^*+/+*^ joints. As *Prg4*^*GT/GT*^ animals aged, their synovia contained higher percentages of CD86+/CD206− and CD86+/CD206+ macrophages and a lower percentage of CD86−/CD206+ macrophages. **H** M1/M2 ratio in synovial tissues from 6-month-old *Prg4*^*GT/GT*^ animals was higher than the corresponding ratio in age-matched *Prg4*^*+/+*^ animals

The study of SRMs was performed according to the scheme presented in Supplementary Figure 1A, and gating of this macrophage population was conducted as shown in Supplementary Figure 2 and Fig. 1A. In addition, the study of total macrophages in the synovial tissues was conducted according to the scheme presented in Supplementary Figure 1A and was identified according to the gating strategy in Supplementary Figure 2 and Fig. 1B. The percentages of total macrophages that were identified as SRMs in 2- and 6-month-old *Prg4*^*GT/GT*^ joints were lower than age-matched *Prg4*^*+/+*^ joints (*p<0.001*; *p<0.0001*; Fig. 1C). In addition, 6-month-old *Prg4*^*GT/GT*^ joints had a lower percentage of SRMs compared to 2-month-old *Prg4*^*GT/GT*^ joints (*p<0.001*; Fig. 1C), whereas *Prg4*^*+/+*^ joints did not show a significant alteration in SRM percentage as animals aged (*p>0.05*). Representative flow cytometry plots showing CX3CR1+ SRMs in *Prg4*^*+/+*^ and *Prg4*^*GT/GT*^ animals are shown in Fig. 1D. The CX3CR1+ fraction of SRMs declined in *Prg4*^*GT/GT*^ animals as they aged. While the percentage of CX3CR1+ SRMs in 2-month-old *Prg4*^*GT/GT*^ animals was higher compared to age-matched *Prg4*^*+/+*^ animals (*p<0.05*; Fig. 1E), CX3CR1+ SRM percentage in 6-month-old *Prg4*^*GT/GT*^ joints was not significantly different from *Prg4*^*+/+*^ joints but was lower than 2-month-old *Prg4*^*GT/GT*^ joints (*p<0.01*). Total macrophages were higher in joints of 2- and 6-month-old *Prg4*^*GT/GT*^ animals compared to *Prg4*^*+/+*^ animals (*p<0.0001*; Fig. 1F). Macrophages in the synovial tissues comprised heterogenous subpopulations with differential expression patterns of CD86 and/or CD206 proteins. CD86+/CD206− and CD86+/CD206+ macrophages were higher in 2- and 6-month-old *Prg4*^*GT/GT*^ joints compared to *Prg4*^*+/+*^ animals (*p<0.05 for all comparisons*; Fig. 1G), while CD86−/CD206+ macrophages were depleted in 6-month-old *Prg4*^*GT/GT*^ joints (*p<0.001*; Fig. 1G). The M1/M2 ratio increased with age in *Prg4*^*GT/GT*^ animals compared to *Prg4*^*+/+*^ animals (Fig. 1H). M1/M2 ratio was higher in 6-month-old *Prg4*^*GT/GT*^ synovia compared to 2-month-old *Prg4*^*+/+*^ and 6-month-old *Prg4*^*+/+*^ synovia (*p<0.001*; *p<0.0001*; Fig. 1H). These findings support that as *Prg4*^*GT/GT*^ animals aged, the total number of macrophages increased, while the fraction of the macrophages that are tissue-resident declined and that change was associated with a shift towards a higher M1/M2 ratio.

**Fig. 2:**
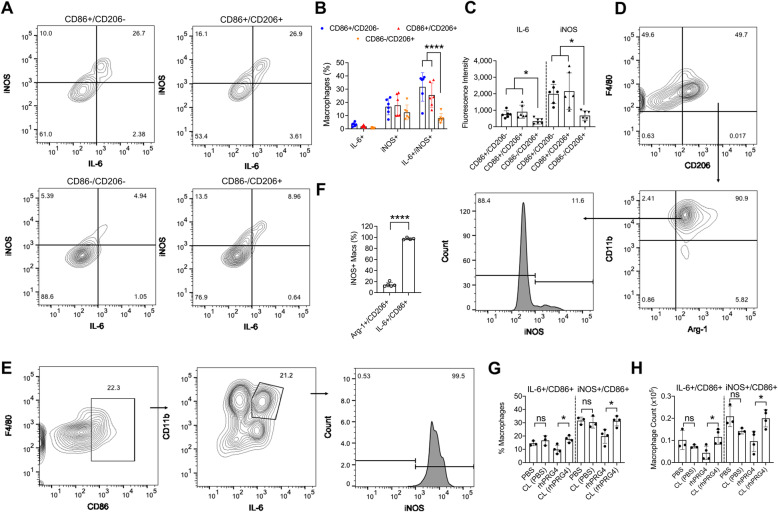
Characterization of the expression of inflammatory markers, interleukin-6 (IL-6), and inducible nitric oxide synthase (iNOS) in gated synovial macrophages that express CD86 and/or CD206 surface markers from 2-month-old *Prg4* gene-trap (*Prg4*^*GT/GT*^) animals and impact of intra-articular (IA) recombinant human proteoglycan-4 (rhPRG4) treatment on IL-6 and iNOS expression in CD86+ macrophages. Macrophages were identified as CD11b+ F4/80+ MHC class II− CD86+ and/or CD206+ and the expression levels of IL-6 and iNOS (both are markers of inflammatory M1 macrophages) and arginase-1 (Arg-1) (a marker of anti-inflammatory M2 macrophages) were evaluated using intracellular staining with PE-anti-IL-6, Alexa Fluor 488-anti-iNOS, and PE-anti-Arg-1 antibodies. Positivity thresholds were set using fluorescence minus one (FMO) panel controls. rhPRG4 (1 mg/mL; 10μL) or phosphate-buffered saline (PBS) (10μL) were administered IA in 2-month-old *Prg4*^*GT/GT*^ animals and analysis of IL-6 and iNOS positivity in CD11b+ F4/80+ MHC class II− CD86+ macrophages was performed at 24 h following IA treatments. Macrophage cell counts were determined using precision counting beads and expressed as numbers per joint. Statistical analyses of IL-6 and iNOS staining intensities in macrophages and percentages of IL-6+ and/or iNOS+ macrophages were performed using one-way and two-way ANOVA, respectively. Tukey’s post hoc test was used in one- and two-way ANOVAs. Analysis of iNOS positivity in Arg-1+/CD206+ and IL-6+/CD86+ macrophages and impact of rhPRG4 treatment was performed using unpaired and paired Student’s *t* test, respectively. Experimental groups included three (2 males and one female) to six animals (3 males and 3 females). In all experiments, independent biological samples were analyzed in duplicates, and the averages of both technical replicates were included in the analysis. ns non-significant; *p<0.05 and ****p<0.0001. **A** A representative flow cytometry contour plot of IL-6 and iNOS probing in CD86+ and/or CD206+ macrophages. Gating for CD86+ and/or CD206+ macrophages was performed according to supplementary figure 2 and fig. 1B and cells in each quadrant were plotted according to their IL-6 and iNOS staining intensities, and percentages of IL-6+, iNOS+, and IL-6+/iNOS+ macrophages were determined. **B** Percentages of dually positive IL-6 and iNOS CD86+/CD206− and CD86+/CD206+ macrophages were higher than dually positive IL-6 and iNOS CD86−/CD206+ macrophages. **C** Mean IL-6 and iNOS staining intensities were higher in CD86+/CD206− and CD86+/CD206+ macrophages compared to CD86−/CD206+ macrophages. **D** Gating strategy to identify Arg-1+/CD206+ macrophages in synovial tissues from 2-month-old *Prg4*^*GT/GT*^ joints. Singlets and viable cells were identified as shown in Supplementary Figure 2. Viable cells were gated according to their CD206 and F4/80 expression status. Dually positive cells were then gated for Arg-1 and CD11b to identify Arg-1+/CD206+ macrophages. The percentage of the iNOS+ subset was subsequently determined. **E** Gating strategy to identify IL-6+/CD86+ macrophages in synovial tissues from 2-month-old *Prg4*^*GT/GT*^ joints. Singlets and viable cells were identified as shown in Supplementary Figure 2. Viable cells were gated according to their CD86 and F4/80 expression status. Dually positive cells were then gated for IL-6 and CD11b to identify IL-6+/CD86+ macrophages. The percentage of the iNOS+ subset was subsequently determined. **F** The percentage of iNOS+/IL-6+/CD86+ macrophages was higher than the corresponding percentage in iNOS+/Arg-1+/CD206+ macrophages. **G** rhPRG4 treatment reduced the percentages of IL-6+/CD86+ and iNOS+/CD86+ macrophages compared to contralateral (CL) joints. No reductions were observed with PBS treatment. **H** rhPRG4 treatment reduced the numbers of IL-6+/CD86+ and iNOS+/CD86+ macrophages compared to contralateral (CL) joints. No reductions were observed with PBS treatment

CD86+ and/or CD206+ macrophages in 2-month-old *Prg4*^*GT/GT*^ joints were further classified according to their iNOS and IL-6 expression status (Fig. 2A). CD86+/CD206− and CD86+/CD206+ macrophages contained higher percentages of IL-6+/iNOS+ cells compared to CD86−/CD206+ macrophages, and the magnitude of this increase was ~ 4.5-fold (*p<0.0001*; Fig. 2B). Similarly, mean IL-6 and iNOS staining intensities were higher in CD86+/CD206− and CD86+/CD206+ macrophages compared to corresponding mean intensities in CD86−/CD206+ macrophages (*p<0.05*; Fig. 2C). We further studied CD206+ macrophages to identify whether they co-expressed classical M2 markers, e.g., Arg-1. We observed that in 2-month-old *Prg4*^*GT/GT*^ animals, a significant proportion of CD206+ macrophages (~90%) was also positive for Arg-1 (Fig. 2D). Arg-1+/CD206+ macrophages did not appreciably express iNOS as approximately 88% of these macrophages were iNOS− (Fig. 2D). In contrast, IL-6+/CD86+ macrophages were also iNOS+ (*p<0.0001*; Fig. 2E & F). The study of the impact of IA rhPRG4 treatment on CD86+ pro-inflammatory macrophages was conducted according to the scheme presented in Supplementary Figure 1C. At 24h, rhPRG4 treatment reduced IL-6+ and iNOS+ CD86+ macrophages in 2-month-old *Prg4*^*GT/GT*^ joints (*p<0.05* for both comparisons; Fig. 2G & H). In contrast, PBS did not alter IL-6 or iNOS expression in CD86+ macrophages (*p>0.05*; Fig. 2G & H).

*Prg4 recombination limited the accumulation of macrophages in Prg4*^*GT/GT*^*joints, populated the synovium with CD86*−*/CD206+ (M2) macrophages, and reduced pro-inflammatory macrophages recruitment in response to TLR2 stimulation.*

**Fig. 3:**
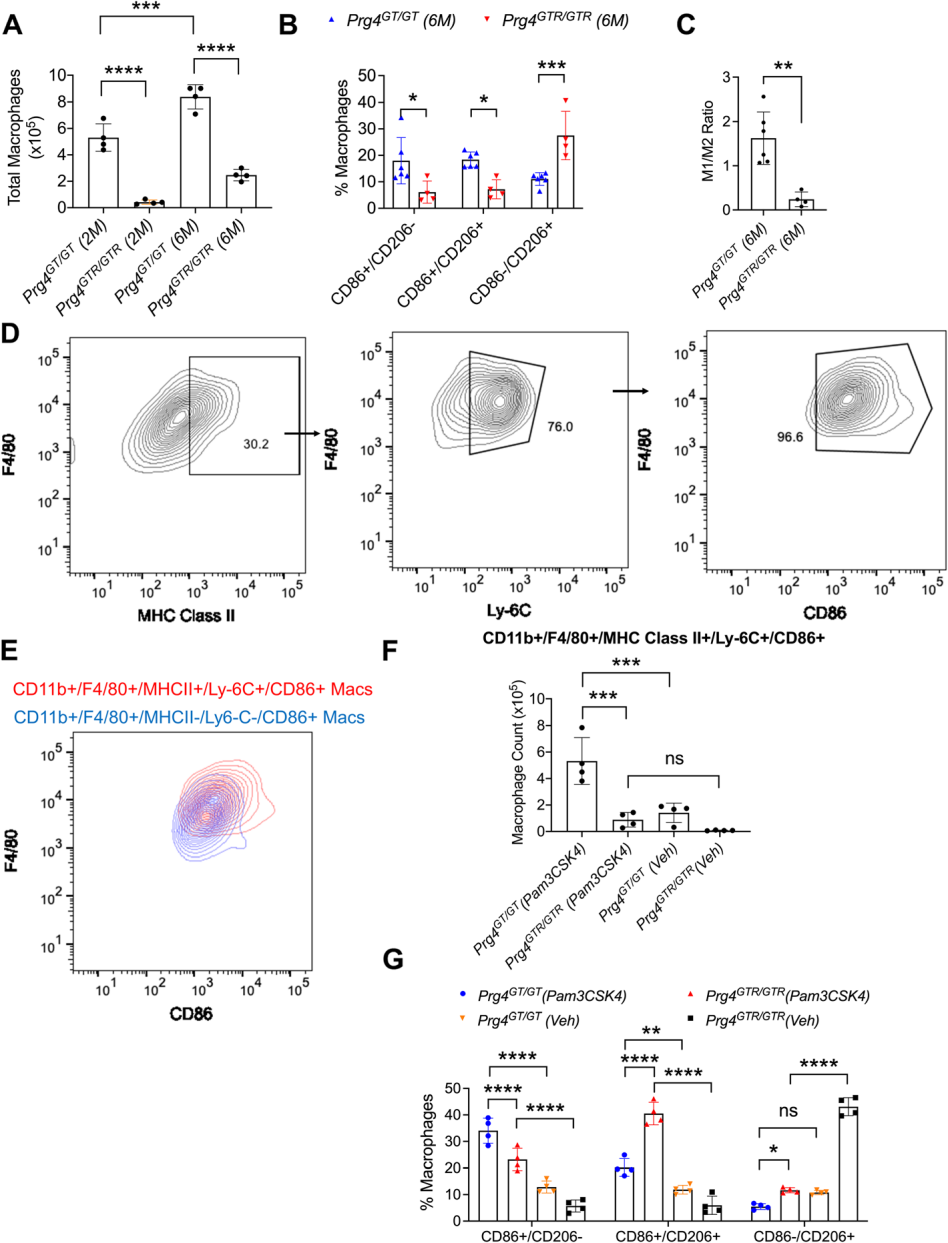
Impact of endogenous *Prg4* recombination on macrophage accumulation and polarization in synovial tissues isolated from the knee joints of *Prg4* gene-trap (*Prg4*^*GT/GT*^) animals and the role of PRG4 in regulating inflammatory macrophage recruitment to the joint in response to Pam3CSK4, a Toll-like receptor 2 (TLR2) agonist. *Prg4* gene recombination (*Prg4*^*GTR/GTR*^) was performed in 3-week-old mice by intraperitoneal administration of tamoxifen (0.1 mg/g) in corn oil vehicle daily for 10 days, using vehicle-only administered age-matched *Prg4*^*GT/GT*^ as controls. Pam3CSK4 (3μg in 10-μL sterile water) or vehicle (10μL) treatments were performed in age-matched 2-month-old *Prg4*^*GT/GT*^ or *Prg4*^*GTR/GTR*^ animals (*n*=4 per group; 2 males and 2 females), and macrophage polarization and newly infiltrated inflammatory macrophage accumulation were determined in synovial tissues at 72h post-injections. Macrophages in 2- and 6-month-old *Prg4*^*GTR/GTR*^ animals were compared to age-matched *Prg4*^*GT/GT*^ animals (*n*=4–6 per group). Macrophages in synovial tissues were identified as CD11b+ F4/80+ MHC class II− CD86+ and/or CD206+ (using gating strategies shown in Supplementary Figure 2 and in Fig. 1B). Newly infiltrated inflammatory macrophages were identified as CD11b+ F4/80+ MHC class II+ Ly-6C+ CD86+. Macrophage cell counts were determined using precision counting beads and expressed as numbers per joint. We compared the ratio of M1 (CD11b+ F4/80+/MHC class II− CD86+/CD206−) and M2 (CD11b+ F4/80+ MHC class II− CD86−/CD206+) macrophages in synovial tissues from 6-month-old *Prg4*^*GT/GT*^ and *Prg4*^*GTR/GTR*^ animals. In all experiments, independent biological samples were analyzed in duplicates, and the averages of both technical replicates were included in the analysis. Statistical analyses of total macrophages and percentages of CD86+ and/or CD206+ macrophages were performed using one-way and two-way ANOVA, respectively. Tukey’s post hoc test was used in one- and two-way ANOVAs. ns non-significant; *p<0.05; **p<0.01; ***p<0.001; and ****p<0.0001. **A** ***P****rg4* gene recombination at 3 weeks reduced macrophage accumulation in 2- and 6-month-old joint tissues. **B** ***P****rg4*^*GTR/GTR*^ joints at 6 months contained lower percentages of CD86+/CD206− and CD86+/CD206+ macrophages and a higher percentage of CD86−/CD206+ macrophages compared to age-matched *Prg4*^*GT/GT*^ joints. **C M**1/M2 ratio was reduced as a consequence of *Prg4* recombination. **D** Gating strategy to identify newly infiltrated inflammatory macrophages. Singlets and viable cells were identified as shown in Supplementary Figure 2. Cells were then gated for CD11b and F4/80 and identified MHC class II+ Ly-6C+ CD86+ population as shown in the representative flow cytometry contour plot. **E** Representative flow cytometry contour plot depicting stronger CD86 epitope staining in newly infiltrated inflammatory macrophages compared to the existing macrophage populations in synovial tissues of Pam3CSK4-administered *Prg4*^*GT/GT*^ joints. **F** Pam3CSK4 administration resulted in greater inflammatory macrophage recruitment in 2-month-old *Prg4*^*GT/GT*^ joints compared to *Prg4*^*GTR/GTR*^ joints. **G** Pam3CSK4 administration resulted in a shift towards an inflammatory CD86+ macrophage, particularly in 2-month-old *Prg4*^*GT/GT*^ joints

The *Prg4* recombination studies were conducted according to the scheme presented in Supplementary Figure 1B. Gating for CD86+ and/or CD206+ macrophages was conducted as shown in Supplementary Figure 2 and Fig. 1B. *Prg4* recombination reduced total macrophages in synovial tissues of 2- and 6-month-old animals (*p<0.0001* for both comparisons; Fig. 3A). In addition, *Prg4* recombination reduced the percentages of CD86+/CD206− and CD86+/CD206+ macrophages compared to age-matched non-recombined animals (*p<0.05* for both comparisons; Fig. 3B). *Prg4* recombination also increased the percentage of CD86−/CD206+ macrophages compared to non-recombined animals (*p<0.001*; Fig. 3B). The modulation of CD86+ and/or CD206+ macrophages by *Prg4* re-expression resulted in a lower M1/M2 ratio in synovia of 6-month-old *Prg4*^*GTR/GTR*^ animals compared to age-matched *Prg4*^*GT/GT*^ animals (*p<0.01*; Fig. 3C).

The gating strategy to identify infiltrated pro-inflammatory macrophages, generated from circulating monocytes (characterized by positive MHC class II and Ly-6C expressions) is shown in Fig. 3D. This macrophage population had an M1 pro-inflammatory phenotype demonstrated by greater CD86 expression compared to the existing macrophage pool in *Prg4*^*GT/GT*^ joints (Fig. 3E). TLR2 stimulation induced the recruitment of higher numbers of pro-inflammatory macrophages in *Prg4*^*GT/GT*^ joints compared to *Prg4*^*GTR/GTR*^ joints (*p<0.001*; Fig. 3F). Furthermore, TLR2 stimulation resulted in a shift towards increased CD86+/CD206− macrophages and reduced CD86−/CD206+ macrophages in *Prg4*^*GT/GT*^ joints compared to *Prg4*^*GTR/GTR*^ joints (*p<0.0001*; *p<0.05*; Fig. 3G).

*TLR2-stimulated M2a BMDMs from Prg4* ^*GT/GT*^ *animals exhibited elevated expressions of inflammatory cytokines and rhPRG4 treatment reduced the secretion of cytokines and chemokines by these BMDMs.*

**Fig. 4:**
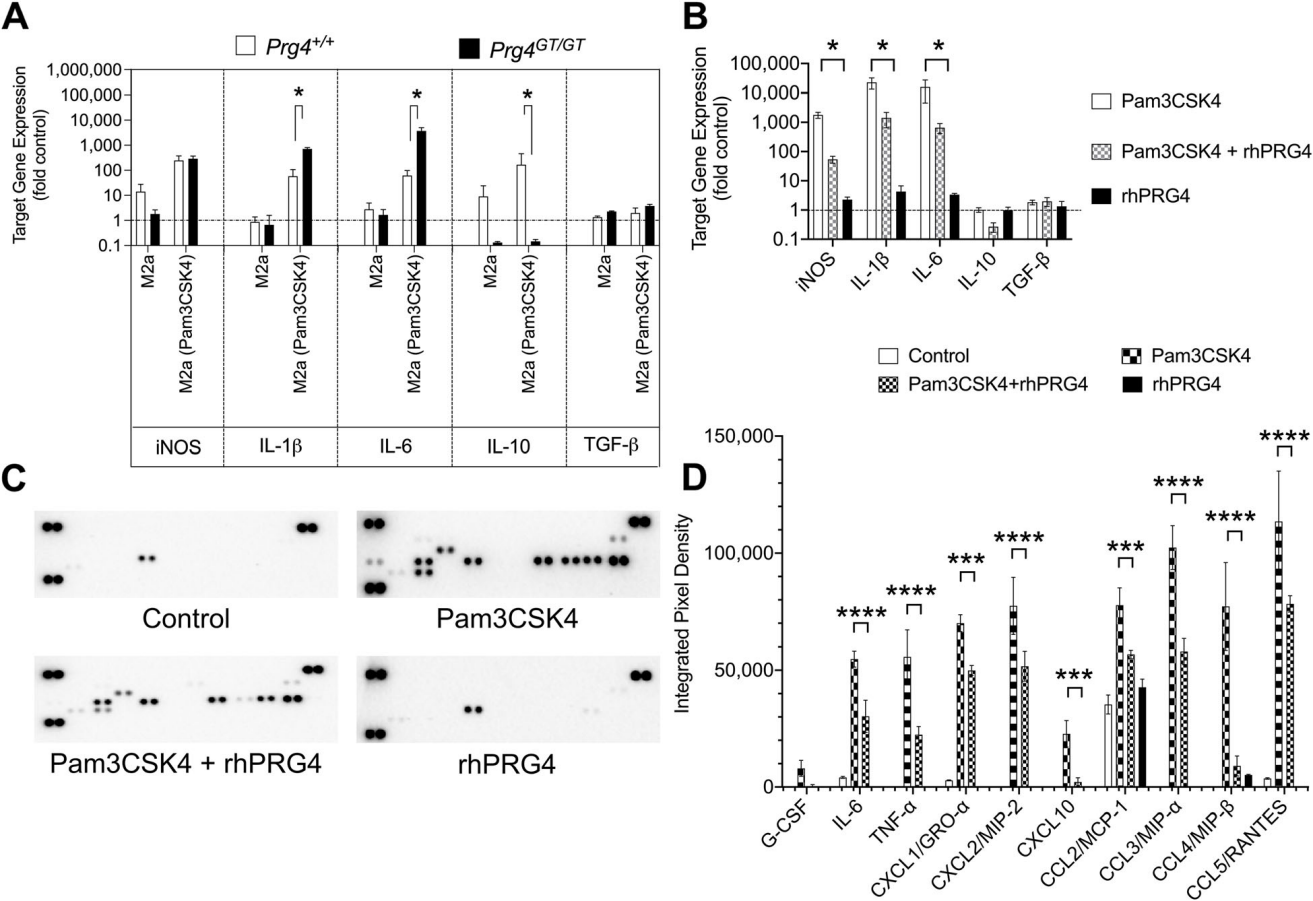
Stimulation of M2a polarized bone marrow-derived macrophages (M2a BMDMs) from 2-month-old *Prg4* gene-trap (*Prg4*^*GT/GT*^) and *Prg4*^*+/+*^ mice by Pam3CSK4, a Toll-like receptor 2 (TLR2) agonist, and efficacy of recombinant human proteoglycan-4 (rhPRG4) in reducing the secretion of cytokines and chemokines in Pam3CSK4-stimulated *Prg4*^*GT/GT*^ M2a BMDMs. M2a polarization was performed using interleukin-4 (IL-4)/IL-13. M2a BMDMs were treated with Pam3CSK4 (100ng/mL) ± rhPRG4 (200μg/mL) for 24h followed by gene expression studies of inducible nitric oxide synthase (iNOS), IL-1β, IL-6, IL-10, and TGF-β. Cytokine and chemokine levels in media supernatants were quantified using a murine cytokine array. The proteome profiler cytokine array panel map is presented in Supplementary Figure 3. Data is presented as mean ± standard deviation of three independent experiments, with cells derived from 2–3 animals per experiment. Analyses of cycle threshold (Ct) values and cytokine/chemokine levels were performed using one-way ANOVA, followed by Tukey’s post hoc test. ns non-significant; *p<0.05; **p<0.01; ***p<0.001; and ****p<0.0001. **A** Pam3CSK4 induced higher IL-1β, IL-6, and lower IL-10 expressions in M2a-polarized BMDMs from *Prg4*^*GT/GT*^ animals compared to BMDMs from *Prg4*^*+/+*^ animals. M0-polarized BMDMs from each genotype were used as controls. **B** rhPRG4 treatment reduced iNOS, IL-1β, and IL-6 expression in Pam3CSK4-stimulated M2a BMDMs. **C** Representative cytokine array blots showing attenuated signal intensities with rhPRG4 treatment. **D r**hPRG4 treatment reduced the secretion of inflammatory cytokines and chemokines by Pam3CSK4-stimulated M2a BMDMs.

M2a BMDMs from *Prg4*^*GT/GT*^ animals had higher expression levels of IL-1β and IL-6 and a lower expression level of IL-10 following TLR2 stimulation (*p<0.05* for all comparisons; Fig. 4A). While TLR2 stimulation induced iNOS expression in BMDMs from *Prg4*^*GT/GT*^ and wildtype animals, there was no difference in iNOS expression levels between the two genotypes (*p>0.05*; Fig. 4A). rhPRG4 treatment reduced TLR2 agonist-induced expression of iNOS, IL-1β, and IL-6 (*p<0.05* for all comparisons) with no effect on IL-10 expression in *Prg4*^*GT/GT*^ M2a BMDMs (Fig. 4B). Representative proteome profiler blots of Pam3CSK4 ± rhPRG4-treated BMDMs showing reducing signal intensities of select cytokines and chemokines are depicted in Fig. 4C. The proteome profiler cytokine array map is shown in Supplementary Figure 3. rhPRG4 treatment reduced secreted levels of inflammatory cytokine: IL-6 (*p<0.0001*) and TNF-α (*p<0.0001*) and chemokines: CXCL1/GRO-α (*p<0.001*), CXCL2/MIP-2 (*p<0.0001*), CXCL10 (*p<0.001*), CCL2/MCP-1 (*p<0.001*), CCL3/MIP-α (*p<0.0001*), CCL4/MIP-β (*p<0.0001*), and CCL5/RANTES (*p<0.0001*) (Fig. 4D).

### Depletion of macrophages reversed synovial fibrosis in Prg4^GT/GT^ knee joints

**Fig. 5:**
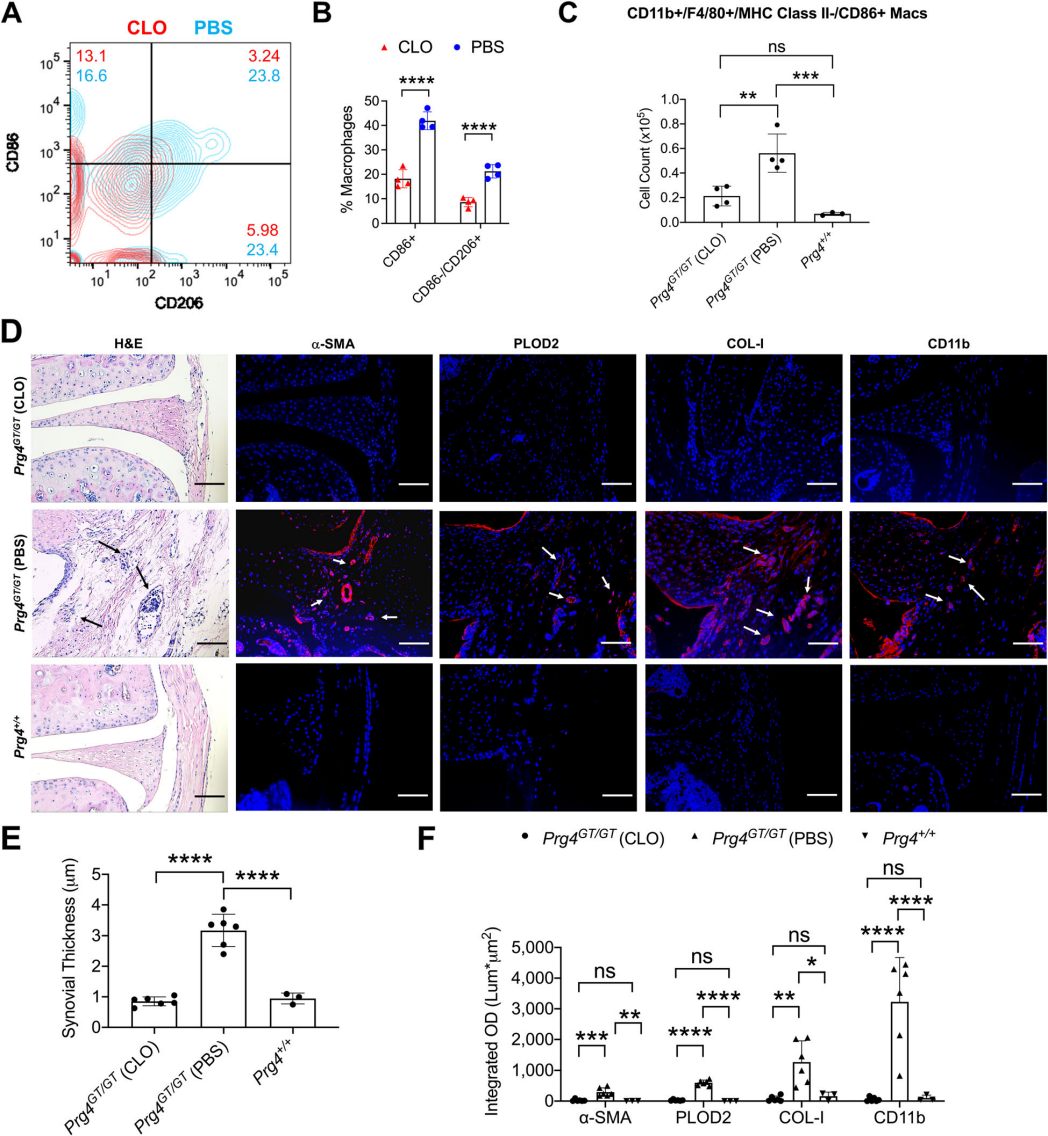
Impact of macrophage depletion on synovial pathology in 4-month-old *Prg4* gene-trap (*Prg4*^*GT/GT*^) animals. The knee joints of *Prg4*^*GT/GT*^ animals were injected with clodronate liposomes (CLO) (10μL; 18.4 mM) or PBS liposomes (PBS) (10μL) twice weekly for 2 weeks according to the scheme presented in Supplementary Figure 1D. The percentages of CD11b+ F4/80+ MHC class II**−** CD86+ or CD86**−**/CD206+ macrophages were determined in cell suspensions from digested joint capsule tissues on day 15 following treatment initiation, and the numbers of CD86+ macrophages were determined using precision counting beads. Cell gating was performed as described in Supplementary Figure 2 and Fig. 1B. In a separate study, synovial pathology measurements were performed on day 30 following treatment initiation and included synovial thickness and staining intensities of fibrosis markers: alpha-smooth muscle actin (α-SMA), PLOD2, and collagen type I (COL-I) as well as the monocyte/macrophage marker CD11b (*n*=6 per group). Age-matched *Prg4* wildtype animals (*Prg4*^*+/+*^, *n*=3) were used as controls. Analyses of macrophage cell count, synovial thickness, α-SMA, PLOD2, COL-I, and CD11b staining intensities were performed using one-way ANOVA followed by Tukey’s post hoc test. Analysis of CD86+ or CD86**−**/CD206+ macrophages was performed using Student’s *t* test. Scale = 10μm. ns non-significant; *p<0.05; **p<0.01; ***p<0.001; and ****p<0.0001. **A** Representative contour plot of CD86 and CD206 staining in macrophages of synovial tissues from CLO- or PBS-treated joints at 15 days following treatment initiation. The most prominent change with CLO treatment was a reduction in the percentage of CD86+/CD206+ macrophages. **B** Percentages of CD86+ and CD86**−**/CD206+ macrophages were reduced with CLO treatment. **C** Inflammatory macrophages in *Prg4*^*GT/GT*^ joints were depleted with CLO treatment. **D** Representative histological sections (H&E, α-SMA, PLOD2, COL-I, and CD11b) depict thickening of the synovial membrane, immune cell infiltration, positive α-SMA, PLOD2, COL-I, and CD11b staining (all indicated by arrows) in PBS-treated joints. Sections were counterstained with DAPI. Macrophage depletion reduced synovial thickness in *Prg4*^*GT/GT*^ joints. **E** Macrophage depletion reduced α-SMA, PLOD2, COL-I, and CD11b staining in *Prg4*^*GT/GT*^ joints

Macrophage depletion studies were conducted according to the scheme presented in Supplementary Figure 1D. Gating of macrophage populations of interest was performed as described in Supplementary Figure 2 and Fig. 1B. In our intermediate endpoint study (conducted on day 15 following treatment initiation), we observed that clodronate liposome treatment altered the distribution of CD86+ and/or CD206+ macrophages in synovial tissues towards a reduction in CD86+ and/or CD206+ fractions, and a reduction in the number of events classified as CD11b+ F4/80+ MHC class II- cells (Fig. 5A), consistent with a depleting effect on synovial macrophages. As a consequence of clodronate liposome treatment, the percentages of CD86+ and CD86−/CD206+ macrophages were reduced compared to PBS liposome treatment (*p<0.0001* for both comparisons; Fig. 5B). As shown above, CD86+ macrophages populated the synovium as animals aged, and as such, they may be causally implicated in the progressive development of synovial hyperplasia and fibrosis. Therefore, we studied the impact of clodronate on CD86+ macrophage numbers in *Prg4*^*GT/GT*^ synovia and detected a reduction that approximated 62% in magnitude (*p<0.01*; Fig. 5C). In our histology endpoint studies (conducted on day 30 following treatment initiation), we observed more significant macrophage depletion demonstrated by fewer immune cells in H&E-stained sections and reduced CD11b staining in synovial tissues of clodronate-treated *Prg4*^*GT/GT*^ joints (*p<0.0001*; Fig. 5D & F). Synovial membrane thickness was also reduced *(p<0.0001)* with clodronate liposomes (Fig. 5E). In addition, no appreciable α-SMA, PLOD2, or COL-I staining was detected in synovial tissues of clodronate liposome-treated *Prg4*^*GT/GT*^ joints, while these markers were detected in synovial tissues of PBS liposomes-treated *Prg4*^*GT/GT*^ joints (*p<0.001*; *p<0.0001*; *p<0.01*; Fig. 5D & F).

## Discussion

In this work, we investigated the temporal changes in tissue-resident and total macrophages in synovial tissues from joints lacking *Prg4* expression and discovered that the tissue-resident fraction of macrophages decreased with age along with a simultaneous increase in the total number of macrophages, indicating that macrophages of a non-resident origin have progressively invaded and populated *Prg4* null synovial tissues. In the *Prg4* null synovium, the totality of macrophages exhibited an age-dependent shift towards the pro-inflammatory M1 phenotype and away from the anti-inflammatory M2 phenotype, and as such, a dramatically high M1/M2 ratio was observed in 6-month-old *Prg4* null synovia that coincided with significant synovial pathology. Early in the development of synovial pathology, synovium-resident macrophages appeared to be enriched in the anti-inflammatory CX3CR1+ SRM subtype, potentially to compensate for the emergence of M1 macrophages in *Prg4*^*GT/GT*^ synovia. However, with age and as synovitis persisted, the CX3CR1+ subset diminished, signaling a compromised endogenous anti-inflammatory role for synovial macrophages. A macrophage population that was positive for both CD86 and CD206 was identified in *Prg4*^*GT/GT*^ synovial tissues, and this population increased with age further supporting that in vivo, macrophages can exist with a high degree of plasticity, where both M1 and M2 markers are expressed by the same cell. For this specific macrophage subtype, it was unclear whether it is predominantly pro- or anti-inflammatory. To investigate this, we probed for other functional M1 markers, e.g., IL-6 and iNOS and concluded that CD86+/CD206+ macrophages have a pro-inflammatory phenotype, indicated by enhanced IL-6 and iNOS expressions, that is comparable to CD86+ only macrophages. It is therefore reasonable to assume that this macrophage population plays an effector role, together with CD86+/CD206− macrophages, in mediating chronic synovitis in *Prg4* null joints. CD86+/CD206+ macrophages might have originated from CD86−/CD206+ counterparts in the synovium. This notion is supported by our observation that in TLR2 agonist-treated *Prg* recombined joints, the reduction in CD86−/CD206+ macrophages was accompanied by a comparable increase in CD86+/CD206+ macrophages, suggesting that M2 macrophages expressed CD86 in response to TLR2 stimulation. This phenomenon has been previously reported in human macrophages where M2 macrophages displayed M1 markers in response to TLR2 stimulation [41], and these macrophages had a pro-inflammatory phenotype as well [41]. The involvement of TLR2 signaling in the generation of CD86+/CD206+ macrophages is also supported by observations that TLR2 activation is a requirement for CD86 upregulation in murine macrophages and subsequent Th1 immune response in vitro and in vivo [42].

*Prg4* recombination re-established synovial macrophage homeostasis as it enriched the synovium with the anti-inflammatory M2 macrophages and prevented M1 macrophage accumulation in the synovium and as such modified the M1/M2 ratio to be in line with the corresponding ratio in age-matched wild type tissues. Exogenous PRG4 administration in the joints lacking *Prg4* expression immediately attenuated the pro-inflammatory M1 macrophage phenotype as it reduced iNOS and IL-6 expressions in CD86+ macrophages, arguing for a direct effect of PRG4 on macrophages. To further explore this assumption, we supplemented our in vivo studies with an in vitro investigation into whether PRG4 can directly modulate macrophage responsiveness to inflammatory stimuli. We detected an impaired anti-inflammatory activity of M2 macrophages from *Prg4* null animals evidenced by a reduced IL-10 and increased IL-1β and IL-6 expressions. Furthermore, rhPRG4 reduced the expression and secretion of inflammatory cytokines by TLR2-stimulated M2a BMDMs. Hence, our in vitro experiments support that PRG4 regulates macrophage activation and that its in vivo homeostatic role in the joint might be facilitated by a direct effect on synovial macrophages. However, the biological effect of PRG4 on synovial fibroblasts in the context of attenuating synovial pathology characteristics of *Prg4* null mice should also be considered. PRG4 exerts direct anti-inflammatory, anti-proliferative, and anti-fibrotic effects on synovial fibroblasts [22, 23], and as such, regulation of synovial fibroblasts is a potential mechanistic component of PRG4’s homeostatic role in the synovium. PRG4 also has a lubricating mechanical role in the joint, and the absence of such a role might contribute to the dysregulated synovial macrophage homeostasis observed in *Prg4* null mice. Nonetheless, PRG4’s effect on macrophages in vivo might be biologically significant, specifically in the context of arresting inflammation and related synovial hyperplasia and fibrosis. Our work with macrophage depletion lends support to the important role of macrophages in regulating synovial pathology in *Prg4* null mice. Independent of PRG4, synovial macrophage depletion reduced synovial hyperplasia and fibrosis consistent with the hypothesis that an aberrant pattern of macrophage polarization in the absence of PRG4 is what mediates chronic synovitis in *Prg4* null joints.

Our understanding of the contribution of macrophages to synovial fibrosis is severely limited. In other tissues, distinct macrophage populations appear to coordinate the initiation, maintenance, and resolution of inflammation and its associated tissue repair [43]. Tissue-resident macrophages maintain tissue immune homeostasis [3, 4] and successful resolution of inflammation requires coordination between the switching of pro-inflammatory macrophages to an anti-inflammatory phenotype and the activation of the tissue-resident macrophage pool to aid in tissue repair [4, 44]. In our *Prg4*^*GT/GT*^ model, inflammation was not adequately resolved, which is rationalized by a diminished anti-inflammatory tissue-resident macrophage population and a lack of differentiation of M1 macrophages to M2 macrophages. The inadequate resolution of chronic inflammation in *Prg4* null joints contributed to dysregulated tissue repair and hence the development of synovial fibrosis that progressed with age [23]. Since a significant fraction of macrophages in the synovium was pro-inflammatory, we aimed to evaluate whether depletion of those pro-inflammatory macrophages alters the trajectory of synovial hyperplasia and fibrosis. We utilized liposomal clodronate to deplete macrophages in the synovium since liposomes facilitate the internalization of the hydrophilic clodronate in phagocytic macrophages where clodronate causes their apoptosis [45]. IA clodronate liposomes have successfully been used in depleting synovial macrophages where macrophage depletion can be seen 7 days following IA administration [45, 46]. Clodronate liposomes were used to deplete macrophages in a murine surgically induced experimental OA model [46, 47]. In one report, a single dose of clodronate liposomes depleted synovial-lining macrophages and reduced MMP-3-mediated cartilage degeneration [46], while in another study, a 3-dose regimen over 9 weeks depleted macrophages across the entire thickness of the synovium and reduced synovitis scores and synovial thickness [47]. In our model, we utilized a 4-dose regimen over 2 weeks and assessed early changes to the macrophage pool in *Prg4*^*GT/GT*^ synovia. Clodronate treatment appeared to reduce the fraction of pro-inflammatory and anti-inflammatory macrophages in the synovium. At the time of our histological analyses, macrophages were depleted from the entire thickness of the synovial tissues along with suppressed fibrotic markers’ expression and reduced synovial hyperplasia. Since clodronate treatment resulted in pan macrophage depletion, it is hard to attribute its resolving effect on fibrosis to the depletion of a specific macrophage population. Nonetheless, it is argued that since the predominant macrophage phenotype in the synovium at the time of clodronate administration is pro-inflammatory, clodronate may have exerted its effect via suppressing synovial inflammation due to a significant depleting effect on pro-inflammatory macrophages in the synovium. Our findings collectively highlight that synovial hyperplasia and fibrosis in joints lacking *Prg4* expression are potentially propagated by an enhanced shift towards pro-inflammatory macrophages and that re-establishing macrophage phenotypic homeostasis either via *Prg4* recombination [23] or via depleting synovial macrophages is efficacious in attenuating synovial pathology.

Circulating monocyte recruitment and differentiation into pro-inflammatory macrophages in the synovium adds to the existing M1 macrophage pool and exacerbates synovitis [12]. In our study, the lack of *Prg4* expression in the synovium enhanced monocyte recruitment and differentiation into pro-inflammatory macrophages, and *Prg4* recombination limited the accumulation of infiltrated macrophages into the joint. The magnitude by which pro-inflammatory macrophages populate the synovium, linked to *Prg4* expression status, may explain how PRG4 regulates the recruitment of monocyte-derived pro-inflammatory macrophages. A larger M1 inflammatory pool in the synovium, as seen in *Prg4* null mice, may generate higher quantities of chemokines in the joint in response to TLR2 stimulation and thus increase monocyte recruitment. While we have not specifically measured the secretion of chemokines in *Prg* null and competent joints, we aimed to study whether PRG4 modulates chemokine secretion by BMDMs in vitro. We observed that BMDMs secrete chemokines of the CXC and CC families [48] to variable degrees. CXC chemokines attract neutrophils, lymphocytes, and monocytes to the synovium [48], while CC chemokines attract monocytes, T cells, and natural killer cells [48]. rhPRG4 reduced, by a biologically significant magnitude, the secretion of multiple chemokines with the greatest effect shown for CCL4. Overall, the magnitude of chemokine reduction with rhPRG4 treatment appeared to be greater with CCL2, CCL3, CCL4, and CCL5 compared to CXCL1 and CXCL2. The ability of rhPRG4 to attenuate chemokine secretion by activated macrophages may provide a plausible explanation of why we observed a reduction in monocyte infiltration following *Prg4* recombination.

It is clear that the dichotomous nature of M1–M2 macrophage polarization is insufficient to adequately describe the multifaceted and complex functions of macrophages in the synovium [49]. However, it remains useful to investigate changes in the phenotypic balance of macrophages according to the broad M1–M2 classification, especially if they can be correlated to structural and pathological changes at the tissue level. We have selected two markers for M1 and M2 macrophages that have been used to study macrophage phenotypic balance in synovial tissues of OA patients and pre-clinical surgically induced OA models [49]. We have also used complementary markers, e.g., IL-6 and iNOS to confirm the pro-inflammatory phenotype of macrophage populations of interest. Arg-1 was also used as a confirmatory marker of the M2 phenotype of CD206+ macrophages in 2-month-old animals where the majority of CD206+ macrophages lacked CD86 expression. The changes in macrophage phenotypic balance that we observed in our gene-trap animals with age trended in a similar direction to what has been reported in the murine DMM model where M1 macrophages (F4/80+ CD86+ CD63−) were increased in the synovium at 6 weeks following DMM surgery [50], and M2 macrophages (F4/80+ CD11c+ CD206+) were reduced at 8 weeks following DMM surgery, along with an increase in iNOS+ macrophages [51]. These DMM synovial macrophage studies in conjunction with our current findings provide a strong motivation to investigate the role of PRG4 in regulating synovial macrophage polarization in OA as the basis for its disease-modifying activity [27, 28]. Our study has a number of limitations. One limitation is that we did not study the impact of macrophage depletion on cartilage health in *Prg4*^*GT/GT*^ joints. In addition, we did not investigate the impact of a joint injury, e.g., DMM on synovial macrophage polarization in *Prg4* null and competent mice. We focused our effort on analyzing macrophages in the synovium and faced technical challenges, and we did not characterize macrophages in other joint compartments, e.g., the synovial fluid. Joint fluid macrophages might have a role in the synovial pathology of *Prg4*^*GT/GT*^ animals. However, macrophages in the synovium, due to their physical proximity, would reasonably exert the greatest influence on synovial hyperplasia and fibrosis. In this study, we were not able to sort out the hybrid M1/M2 (CD86+ CD206+) macrophage population in high-enough yield to perform an extensive comparative phenotypic characterization to M1 (CD86+ CD206−) and M2 (CD86− CD206+) macrophages. Finally, we did not compare the extent of IL-6 and iNOS expressions in M1 macrophages from *Prg4*^*GT/GT*^ animals to those in M1 macrophages from wild-type animals following a traumatic joint insult.

## Conclusion

In summary, we have shown that PRG4 plays an important role in maintaining synovial macrophage homeostasis, where a lack of *Prg4* expression increases total macrophage numbers in the synovium, with a pattern of abundant pro-inflammatory macrophages and diminished anti-inflammatory macrophages. As pro-inflammatory macrophages populate the synovium, structural changes, e.g., synovial hyperplasia and fibrosis, become significant and macrophage depletion reverses this synovial pathology. Given that an imbalance between pro-inflammatory and anti-inflammatory synovial macrophages has been reported in patients with OA and pre-clinical OA models, novel treatments that target macrophage polarization to re-establish homeostasis either directly or indirectly via synovial PRG4 upregulation may prove beneficial in treating chronic synovitis and thus slowing OA progression.

## Supplementary Information

**Additional file 1: Supplementary Figure 1.** Overview of experimental design for *in vivo* studies. In our experiments, we pooled joint capsular tissues from two knee joints and identified this sample as an independent biological sample. In experiments where knee joints did not receive a treatment, the right and left knee joints were pooled from the same animal to generate an independent biological replicate. *In these experiments, the “n” refers to independent biological replicates generated by pooling tissues from two animals. Unless otherwise specified, each experimental group contained equal numbers of males and females.

**Additional file 2: Supplementary Figure 2.** Gating strategy to identify singlets and viable cells using Zombie Violet viability dye. Total cells were gated based on forward scatter area (FSC-A) and side scatter area (SSC-A). Subsequently, singlets were identified based on a combination of FSC-A and forward scatter height (FSC-H). Viable cells were identified using Zombie Violet viability dye.

**Additional file 3: Supplementary Figure 3.** Proteome Profiler Mouse Cytokine Array Panel A Map. Ref. Spot: Reference Spot; Neg. Control: Negative Control (PBS).

## Data Availability

Not applicable

## Abbreviations

α-SMA: Alpha smooth muscle actin
ANOVA: Analysis of variance
CD44: Cluster determinant 44
COL-1: Collagen type-1
Ct: Cycle threshold
CX3CR1: C–X3–C motif chemokine receptor 1
DAMPs: Damage-associated molecular patterns
DMEM: Dulbecco’s modified Eagle’s medium
DMM: Destabilization of the medial meniscus
ELISA: Enzyme-linked immunosorbent assay
GAPDH: Glyceraldehyde-3-phosphate dehydrogenase
IL-1β: Interleukin-1 beta
IL-1Ra: Interleukin-1 receptor antagonist
IL-6: Interleukin-6
IL-10: Interleukin-10
iNOS: Inducible nitric oxide synthase
MCP-1: Monocyte chemoattractant protein-1
MHC class II: Major histocompatibility complex class II
MMPs: Matrix metalloproteinases
NF-κB: Nuclear factor kappa b
OA: Osteoarthritis
PBS: Phosphate-buffered saline
PCR: Polymerase chain reaction
PLOD2: Procollagen-lysine, 2-oxogluterate 5-dioxygenase 2
PRG4: Proteoglycan-4
PTOS: Posttraumatic osteoarthritis
RA: Rheumatoid arthritis
rhPRG4: Recombinant human PRG4
SRMs: Synovium-resident macrophages
SM: Synovial macrophages
TLR2: Toll-like receptor 2
TLR4: Toll-like receptor 4
TGF-β1: Transforming growth factor beta 1
