# Correction to: Proteoglycan-4 is an essential regulator of synovial macrophage polarization and inflammatory macrophage joint infiltration

**DOI:** 10.1186/s13075-021-02640-6

**Published:** 2021-10-29

**Authors:** Marwa Qadri, Gregory D. Jay, Ling X. Zhang, Tannin A. Schmidt, Jennifer Totonchy, Khaled A. Elsaid

**Affiliations:** 1 Department of Pharmacology, College of Pharmacy, Jazan University, Jazan, 82826 Kingdom of Saudi Arabia; 2 Department of Emergency Medicine, Rhode Island Hospital, Providence, RI USA; 3 Biomedical Engineering Department, School of Dental Medicine, University of Connecticut, Farmington, CT USA; 4 Department of Biomedical and Pharmaceutical Sciences, Chapman University School of Pharmacy, Rinker Health Sciences Campus, 9401 Jeronimo Road, Irvine, CA 92618 USA

**Correction to: Arthritis Res Ther 23, 241 (2021)**

**https://doi.org/10.1186/s13075-021-02621-9**

Following publication of the original article [1], the authors reported an error as follows:

In the Authors information section, Holly Richendrfer who is not an author on the manuscript was removed. The corrected author information is listed below:

Marwa Qadri, Pharm.D., Ph.D.: Assistant Professor of Pharmacology, Jazan University School of Pharmacy, Jazan, Kingdom of Saudi Arabia.

Gregory D. Jay, MD, Ph.D.: Professor, Emergency Medicine and Engineering, Brown University, Providence, RI, USA.

Ling Zhang, MD: Senior Research Assistant, Rhode Island Hospital, Providence, RI, USA.

Tannin A. Schmidt, Ph.D.: Associate Professor of Biomedical Engineering, University of Connecticut Health Center, Farmington, CT, USA.

Jennifer Totonchy, Ph.D.: Assistant Professor of Biomedical and Pharmaceutical Sciences, Chapman University, Irvine, CA, USA.

Khaled A. Elsaid, Pharm. D, Ph.D.: Associate Professor of Biomedical and Pharmaceutical Sciences, Chapman University, Irvine, CA, USA.
