# Supplementary figures and images for: Proteoglycan-4 is an essential regulator of synovial macrophage polarization and inflammatory macrophage joint infiltration
Source: Arthritis Res Ther. 2021 Sep 14;23:241. doi: 10.1186/s13075-021-02621-9 (PMC8439011; doi:10.1186/s13075-021-02621-9)

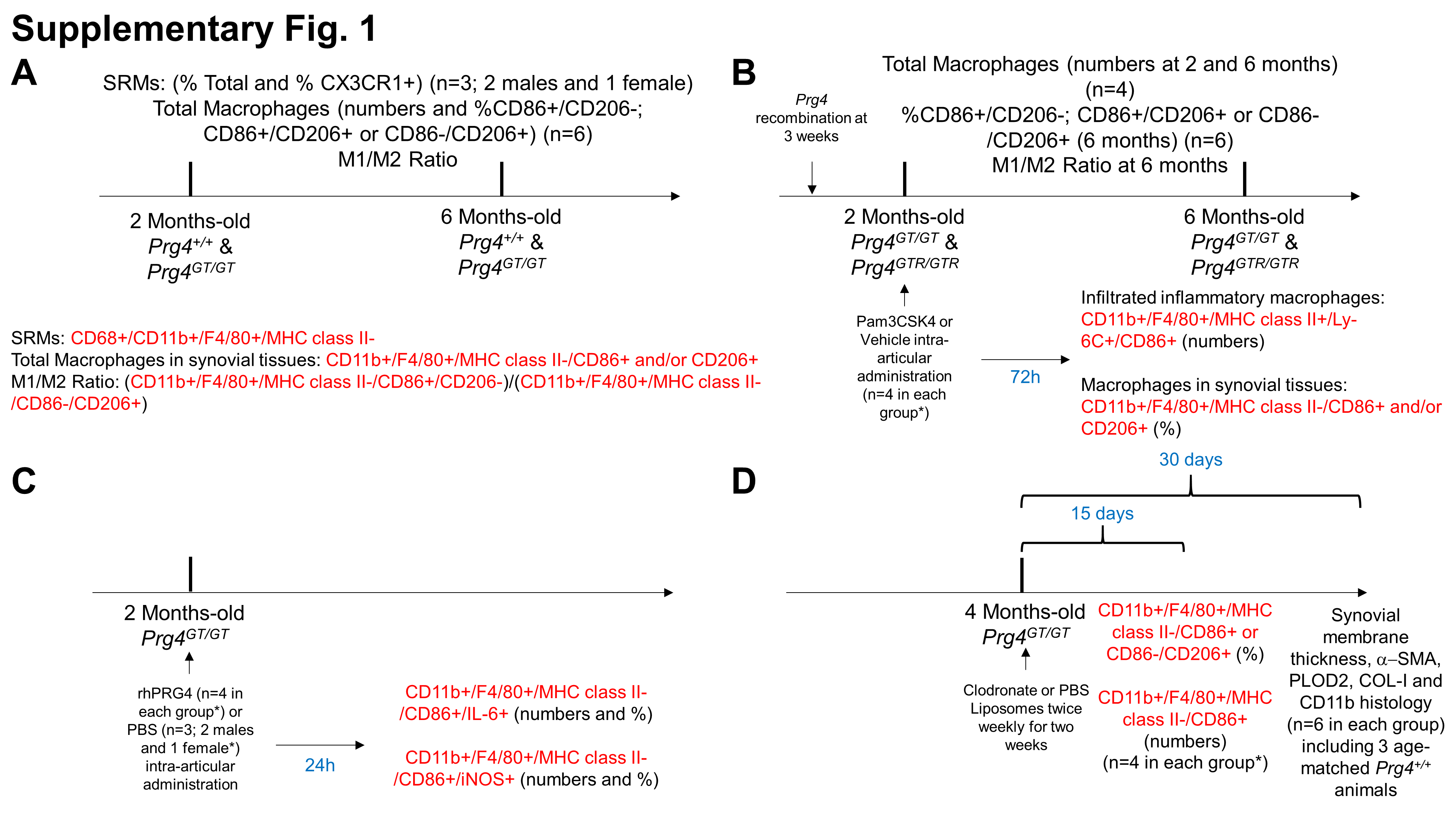

Supplement: Supplementary file 1 — Additional file 1: Supplementary Figure 1. Overview of experimental design for in vivo studies. In our experiments, we pooled joint capsular tissues from two knee joints and identified this sample as an independent biological sample. In experiments where knee joints did not receive a treatment, the right and left knee joints were pooled from the same animal to generate an independent biological replicate. *In these experiments, the “n” refers to independent biological replicates generated by pooling tissues from two animals. Unless otherwise specified, each experimental group contained equal numbers of males and females. [file 13075_2021_2621_MOESM1_ESM.tif]

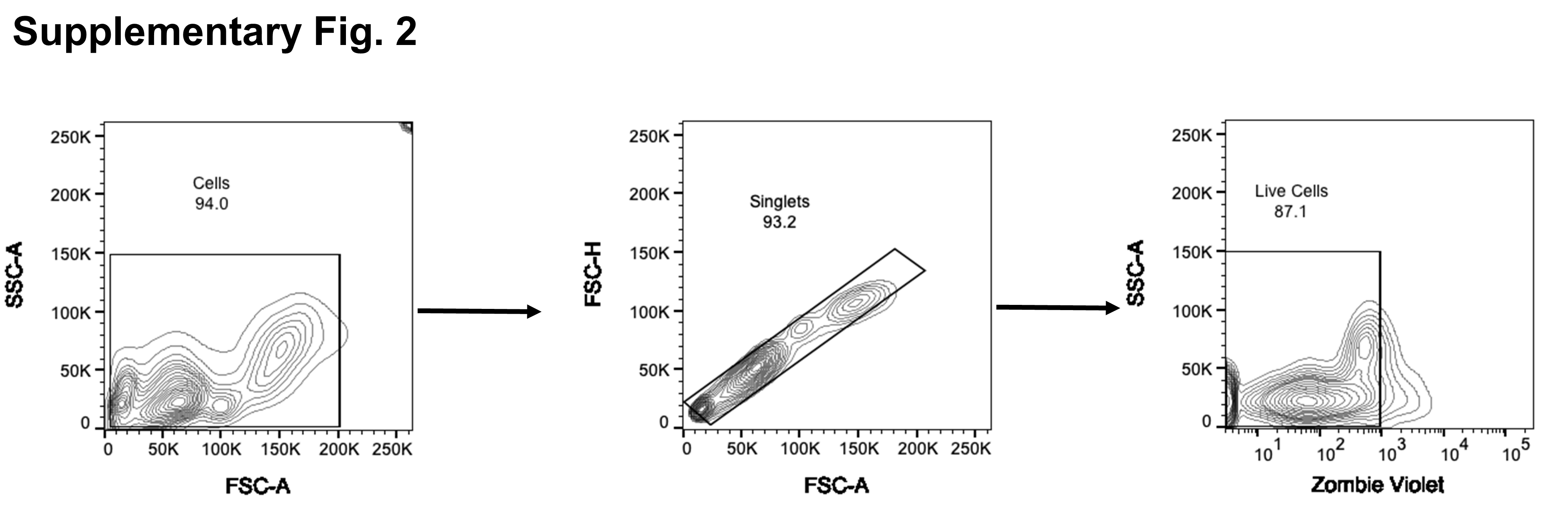

Supplement: Supplementary file 2 — Additional file 2: Supplementary Figure 2. Gating strategy to identify singlets and viable cells using Zombie Violet viability dye. Total cells were gated based on forward scatter area (FSC-A) and side scatter area (SSC-A). Subsequently, singlets were identified based on a combination of FSC-A and forward scatter height (FSC-H). Viable cells were identified using Zombie Violet viability dye. [file 13075_2021_2621_MOESM2_ESM.tif]

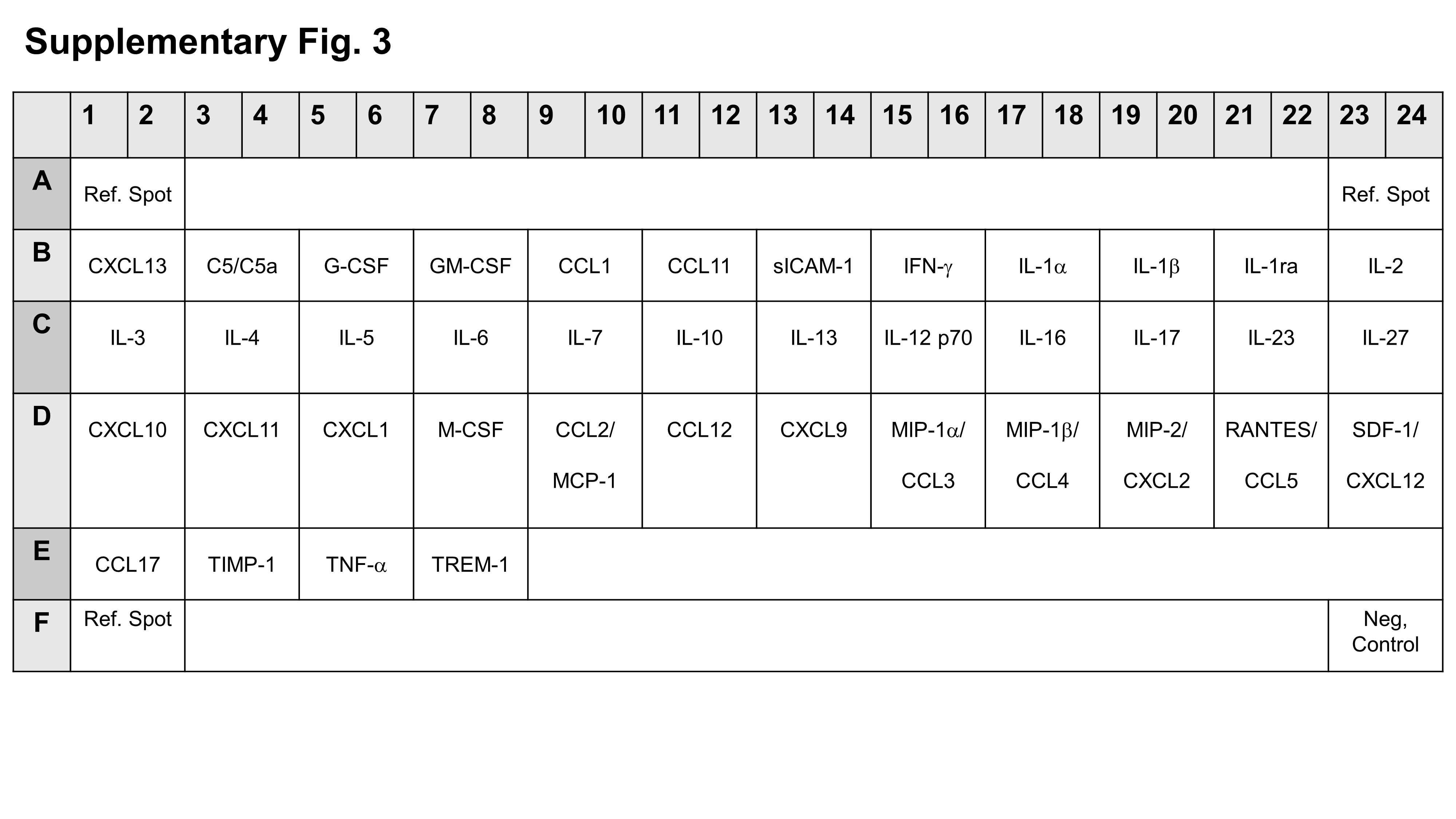

Supplement: Supplementary file 3 — Additional file 3: Supplementary Figure 3. Proteome Profiler Mouse Cytokine Array Panel A Map. Ref. Spot: Reference Spot; Neg. Control: Negative Control (PBS). [file 13075_2021_2621_MOESM3_ESM.tif]
